# Clinical and Molecular Characterization of *SMAD4* Splicing Variants in Patients with Juvenile Polyposis Syndrome

**DOI:** 10.3390/ijms25147939

**Published:** 2024-07-20

**Authors:** Giovanna Forte, Antonia Lucia Buonadonna, Candida Fasano, Paola Sanese, Filomena Cariola, Andrea Manghisi, Anna Filomena Guglielmi, Martina Lepore Signorile, Katia De Marco, Valentina Grossi, Vittoria Disciglio, Cristiano Simone

**Affiliations:** 1 Medical Genetics, National Institute of Gastroenterology-IRCCS “Saverio de Bellis” Research Hospital, 70013 Castellana Grotte, Italy; giovanna.forte@irccsdebellis.it (G.F.); lucia.buonadonna@irccsdebellis.it (A.L.B.); candida.fasano@irccsdebellis.it (C.F.); paola.sanese@irccsdebellis.it (P.S.); filo.cariola@irccsdebellis.it (F.C.); andrea.manghisi@irccsdebellis.it (A.M.); floranna.guglielmi@irccsdebellis.it (A.F.G.); martina.leporesignorile@irccsdebellis.it (M.L.S.); katia.demarco@irccsdebellis.it (K.D.M.); valentina.grossi@irccsdebellis.it (V.G.); 2 Medical Genetics, Department of Precision and Regenerative Medicine and Jonic Area (DiMePRe-J), University of Bari Aldo Moro, 70124 Bari, Italy

**Keywords:** juvenile polyposis syndrome, *SMAD4*, variant of uncertain significance, splicing variants

## Abstract

Juvenile polyposis syndrome (JPS) is an inherited autosomal dominant condition that predisposes to the development of juvenile polyps throughout the gastrointestinal (GI) tract, and it poses an increased risk of GI malignancy. Germline causative variants were identified in the *SMAD4* gene in a subset (20%) of JPS cases. Most *SMAD4* germline genetic variants published to date are missense, nonsense, and frameshift mutations. *SMAD4* germline alterations predicted to result in aberrant splicing have rarely been reported. Here, we report two unrelated Italian families harboring two different *SMAD4* intronic variants, c.424+5G>A and c.425-9A>G, which are clinically associated with colorectal cancer and/or juvenile GI polyps. In silico prediction analysis, in vitro minigene assays, and RT-PCR showed that the identified variants lead to aberrant *SMAD4* splicing via the exonization of intronic nucleotides, resulting in a premature stop codon. This is expected to cause the production of a truncated protein. This study expands the landscape of *SMAD4* germline genetic variants associated with GI polyposis and/or cancer. Moreover, it emphasizes the importance of the functional characterization of *SMAD4* splicing variants through RNA analysis, which can provide new insights into genetic disease variant interpretation, enabling tailored genetic counseling, management, and surveillance of patients with GI polyposis and/or cancer.

## 1. Introduction

Juvenile polyposis syndrome (JPS, OMIM 174900) is a rare autosomal dominant inherited cancer predisposition syndrome, with an estimated incidence varying from 1:16,000 to 1:100,000 [1]. It is characterized by the occurrence of multiple juvenile polyps in the upper and lower gastrointestinal (GI) tract, with a 9–50% increased lifetime risk of developing colorectal (CRC) and gastric cancer (GC) [2]. The diagnosis of JPS is suspected when one or more of the following clinical criteria are met: (i) at least five juvenile polyps in the colon or rectum; (ii) juvenile polyps throughout the GI tract; and (iii) any number of juvenile polyps in an individual with a family history of JPS [3]. JPS polyps vary in size, shape (sessile to pedunculated lesions), and number (from 1 to more than 100). Histologically, juvenile polyps are characterized by cystically dilated crypts with mucus-filled glands, prominent lamina propria, and inflammatory cell infiltration [4].

Germline loss-of-function alterations involving one of two tumor suppressor genes playing a pivotal role in the transforming growth factor-β and bone morphogenetic protein (TGF-β/BMP) signal transduction pathway, namely bone morphogenetic protein receptor type 1A (*BMPR1A*) and SMAD family member 4 (*SMAD4*), are known to be a molecular pathogenic mechanism in JPS [5].

Misregulation of the TGF-β/BMP signaling pathway plays a key role in carcinogenesis since it is involved in the regulation of different cellular mechanisms, including cell cycle progression, cell differentiation, cell death, and cell migration [6].

Overall, germline causative variants in *SMAD4* and *BMPR1A* account for about 45–55% of cases of individuals with one or more clinical criteria of JPS, suggesting that genetic variants in other genes may contribute to JPS etiology.

JPS is characterized by a great variability of clinical manifestations in terms of number, location, and histology of GI polyps, extra-intestinal manifestations, and age at onset [7,8]. Moreover, JPS has incomplete penetrance, and approximately 20–60% of patients with JPS have no family history [5,9,10].

The vast majority of germline alterations affecting the *SMAD4* gene consist of missense, nonsense, or frameshift variants, while germline *SMAD4* large genomic deletions or *SMAD4* alterations predicted to result in aberrant splicing have rarely been reported [11].

Germline characterization of JPS-related predisposing gene variants is extremely important for genetic counseling, clinical management, and tailored surveillance. In this study, we clinically and molecularly characterized two aberrant splicing variants, c.424+5G>A and c.425-9A>G, which were identified in two unrelated Italian families. Using various mRNA analysis approaches, we provide evidence of the pathogenicity of the identified *SMAD4* splicing variant.

Moreover, we expanded our investigation by performing a literature review to explore all molecularly characterized *SMAD4* aberrant splicing variants associated with the JPS clinical phenotype and cancer susceptibility.

## 2. Results

### 2.1. Clinical History and Genetic Findings

**Figure 1:**
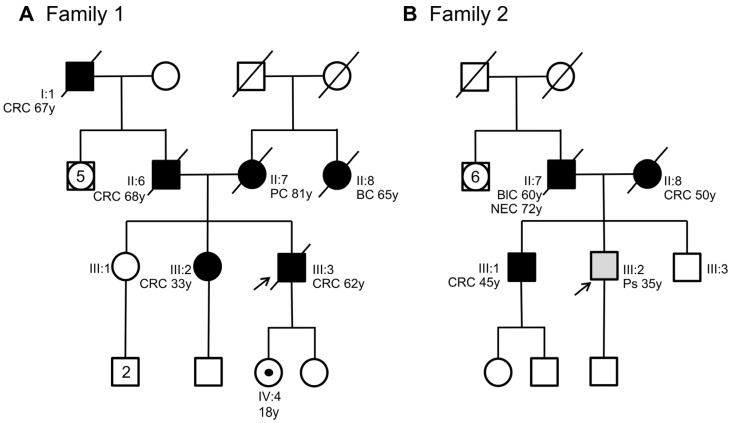
Pedigree of the two families involved in this study. (**A**) Family 1 with members carrying the *SMAD4* c.424+5G>A variant. (**B**) Family 2 with members carrying the *SMAD4* c.425-9A>G variant. Squares indicate men and circles women. The arrow indicates the index case. Unfilled symbols indicate unaffected individuals. A central dot in a symbol denotes an asymptomatic variant carrier. Slashed symbols denote dead individuals. Black-filled symbols denote individuals with cancer, while grey-filled symbols correspond to patients with polyps. The following clinical manifestations are noted below each filled symbol: (CRC = colorectal cancer; PC = pancreatic cancer; BC = breast cancer; BlC = bladder cancer; NEC = neuroendocrine cancer; Ps = polyps), age at diagnosis (y = years).

This study analyzes two unrelated non-consanguineous Italian pedigrees involving seven patients diagnosed with polyposis and CRC. In Family 1, the index case (Figure 1A, III:3) was a man who was diagnosed with and died of CRC at 62 years of age. The personal and familial clinical history of the index case was retrieved from our unit of medical genetics when the patient was already deceased.

**Figure 2:**
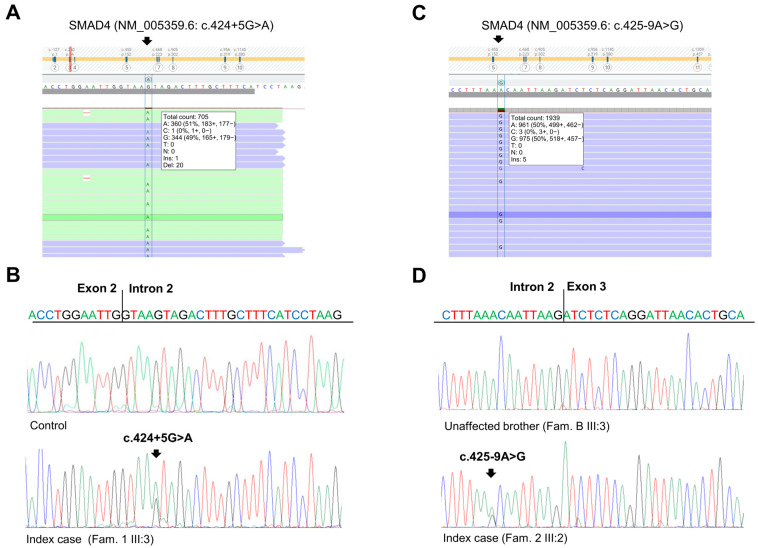
Identification of *SMAD4* germline variants. (**A**) Next-generation sequencing results showing the *SMAD4* c.424+5G>A variant in the index case of Family 1. (**B**) Sequencing electropherograms of genomic DNA from a healthy control individual and the index case of Family 1, confirming the *SMAD4* c.424+5G>A substitution. (**C**) Next-generation sequencing results showing the *SMAD4* c.425-9A>G variant in the index case of Family 2. (**D**) Sequencing electropherograms of genomic DNA from an unaffected member and the index case of Family 2, confirming the *SMAD4* c.425-9A>G substitution.

The patient had a positive family history of cancer. His sister (Figure 1A, III:2), his father (Figure 1A, II:6), and his paternal grandfather (Figure 1A, I:1) developed CRC at 33, 68, and 67 years of age, respectively. The sister underwent genetic analyses of the *MUTYH*, *APC*, *MLH1*, *MSH2*, and *MSH6* genes at another medical genetics center. This analysis did not reveal any germline alteration affecting one of these genes. Moreover, his mother (Figure 1A, II:7) died of pancreatic cancer at 81 years of age, and his maternal aunt died of breast cancer at 65 years of age. Considering the personal clinical history of CRC of the index case and his family history, including three relatives with CRC, the suspicion of hereditary CRC syndrome was raised. Next-generation sequencing (NGS) analysis using a panel of 25 hereditary cancer-related genes performed on the index case’s DNA identified a heterozygous variant in *SMAD4* intron 2 (NM_005359.6: c.424+5G>A) (Figure 2A), which was confirmed by Sanger sequencing (Figure 2B).

This variant was also found in the index case’s daughter who was 18 years old. The patient was immediately referred for clinical surveillance of the organs (stomach and colon) at risk for cancer (Figure 1A, IV:4). The other family members did not agree to undergo *SMAD4* genetic analysis. To date, no other family members have undergone genetic testing.

In Family 2, the index case (Figure 1B, III:2) was a 35-year-old man with a pedunculated hamartomatous polyp and two small hyperplastic polyps in the large intestine. The patient had a positive family history for cancer and polyposis. His brother (Figure 1B, III:1) developed CRC at 45 years of age, while his father (Figure 1B, II:7) developed bladder cancer at 60 years and was diagnosed with and died of neuroendocrine cancer at 72 years of age. Moreover, the index case’s mother (Figure 1B, II:8) developed CRC and numerous polyps at 50 years of age. Histologically, these polyps were classified as likely hamartomatous, juvenile, and adenomatous, with a tubulovillous aspect and moderate or severe dysplasia. Interestingly, the index case’s mother carried a pathogenic variant in *APC* exon 15 (NM_00038.5: c.3336_3340del, p.Asn1113Serfs*4), which was previously identified by a different genetic diagnostic laboratory. This *APC* pathogenic variant was not detected in the index case nor in his CRC-affected brother (Figure 1B, III:2, III:1).

Considering the negative results of the *APC* genetic testing and the clinical manifestation in the index case and his brother, the clinical suspicion of an additional concurrent hereditary colon cancer predisposition syndrome in the Family 2 was advanced.

For these reasons, we expanded the genetic testing of the index case’s DNA by using a panel of 25 hereditary cancer-related genes. This NGS analysis revealed a heterozygous variant in *SMAD4* intron 2 (NM_005359.6: c.425-9A>G) (Figure 2C), which was confirmed by Sanger sequencing (Figure 2D). This variant was also identified in the index case’s mother (Figure 1B, II:8) and CRC-affected brother (Figure 1B, III:1) but not in his healthy brother (Figure 1B, III:3).

The two identified *SMAD4* variants were found to be rare in global population databases (1000 Genome, dbSNP, gnomAD, NHLBI ESP) [12,13,14,15]. Specifically, the *SMAD4* c.425+5G>A variant was reported to have a global minor allele frequency (MAF) of less than 0.01, while the *SMAD4* c.425-9A>G variant was not listed in these databases. In silico analysis using four splice site prediction algorithms (Splice Site Finder (SSF), MaxEntScan (MES), Splice Site Prediction by Neural Network (NNS), and Gene Splicer (GS)) integrated into Alamut VisualPlus version 1.7.2 (Sophia Genetics SAS; Bidart, France) revealed that the *SMAD4* c.424+5G>A and c.425-9A>G variants may result in a splicing defect due to the abrogation of the canonical splice acceptor and donor sites at positions c.424 and c.425 of the *SMAD4* gene, respectively (Figure S1A,B).

### 2.2. Analysis of mRNA by In Vitro Minigene Assay

**Figure 3:**
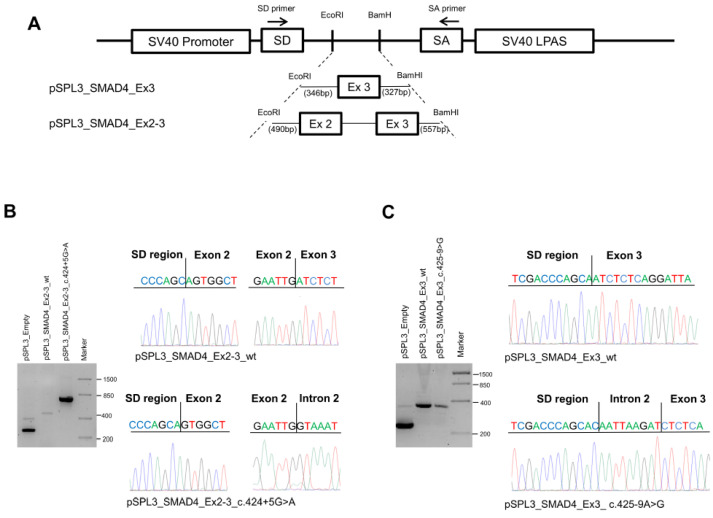
Characterization of *SMAD4* variants. (**A**) Schematic representation of the pSPL3 constructs and localization of the primers (indicated as arrows) used for RT-PCR experiments. (**B**) Agarose gel electrophoresis showing the RT-PCR products obtained in HEK-293 cells transfected with the pSPL3 empty vector (265 bp) or the pSPL3_SMAD4_Ex2-3_wt (467 bp) or pSPL3_SMAD4_Ex2-3_c.424+5G>A (about 800 bp) constructs (left). Sequencing electropherograms of the RT-PCR products originated from the wild-type and mutant cDNA splicing isoforms (right). (**C**) Agarose gel electrophoresis showing the RT-PCR products obtained from HEK-293 cells transfected with the pSPL3 empty vector or the pSPL3_SMAD4_Ex3_wt (295 bp) or pSPL3_SMAD4_Ex3_c.4245-9>G (302 bp) constructs (left). Sequencing electropherograms of the RT-PCR products originated from the wild-type and mutant cDNA splicing isoforms (right).

To determine the effect of the *SMAD4* c.424+5G>A and c.425-9A>G substitutions, we performed minigene assays using pSPL3 plasmid constructs (Figure 3A).

HEK-293 cells were transfected with the empty vector or with the pSPL3_SMAD4_Ex2-3_wt, pSPL3_SMAD4_Ex2-3_c.424+5G>A, pSPL3_SMAD4_Ex3_wt, or pSPL3_SMAD4_Ex3_c.425-9A>G constructs for 36 h; then, total RNA was extracted and amplified by RT-PCR using specific primers. Amplification of the pSPL3_SMAD4_Ex2-3_wt construct resulted in a 467 bp fragment containing exons 2 and 3, while amplification of the pSPL3_SMAD4_Ex2-3_c.424+5G>A construct produced a larger fragment of about 800 bp due to intron retention. The PCR products obtained were confirmed by Sanger sequencing (Figure 3B).

On the other hand, amplification of the pSPL3_SMAD4_Ex3_wt construct resulted in a 295 bp fragment containing exon 3, whereas amplification of the pSPL3_SMAD4_Ex3_c.425-9A>G construct produced a larger fragment (303 bp) due to the retention of the 8-nucleotide CAATTAAG sequence before the exon 3 splice donor, as confirmed by Sanger sequencing (Figure 3C).

### 2.3. Analysis of Patients’ Processed Transcripts

**Figure 4:**
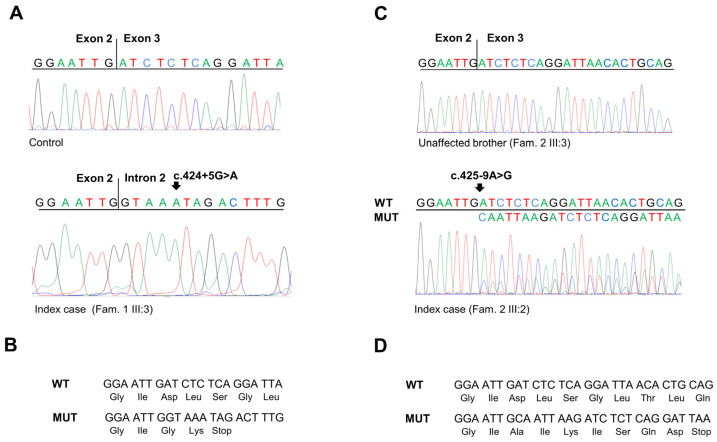
(**A**) Sequencing electropherograms showing the *SMAD4* RT-PCR products obtained from the mRNA extracted from peripheral blood of a healthy control individual and of the index case of Family 1 carrying the *SMAD4* c.424+5 G>A variant. (**B**) Predicted translation products from the *SMAD4* mutated allele with c.424+5 G>A variant (MUT) compared to the *SMAD4* wild-type allele (WT). (**C**) Sequencing electropherograms showing the *SMAD4* RT-PCR products obtained from the mRNA extracted from peripheral blood of an unaffected member and of the index case of Family 2 carrying the *SMAD4* c.425-9A>G variant. (**D**) Predicted translation products from the *SMAD4* mutated allele with c.425-9 A>G variant (MUT) compared to the *SMAD4* wild-type allele (WT).

To corroborate the results obtained in the minigene assays, the effect of the *SMAD4* c.424+5G>A and c.425-9A>G substitutions was further analyzed in the identified families. To this end, total RNA was isolated from peripheral blood of both carrier (Figure 1A, IV:4; Figure 1B, III:2) and non-carrier individuals. Then, specific *SMAD4* exon transcripts were amplified by RT-PCR, and the obtained fragments were sequenced (Figure 4A,C).

Amplification and sequencing of the *SMAD4* exon 2–3 transcript confirmed that the c.424+5G>A substitution results in the exonization of the intronic sequence after the exon 2 splice donor site. This splicing alteration causes a frameshift in the coding region and the creation of a premature stop codon (p.Asp142Glyfs*2) (Figure 4A,B).

Similarly, amplification and sequencing of the *SMAD4* exon 3 transcript confirmed that the c.425-9A>G substitution results in the insertion of eight nucleotides (CAATTAAG) before the canonical splice donor sequence in exon 3, making it a weak splicing signal. Moreover, this generates a new upstream splice donor site that produces a frameshift in the coding region and the creation of a premature stop codon (p.Asp142Alafs*7) (Figure 4C,D).

### 2.4. Literature Review of SMAD4 Splicing Variants

Next, we performed a literature review of *SMAD4* splicing variants to explore the association between genetic alterations that affect *SMAD4* splicing and the JPS clinical phenotype and cancer susceptibility. So far, 12 unique *SMAD4* splicing variants, including those characterized in the present study, have been described in 24 patients with JPS or a personal and familial history of cancer (Figure S2, Table S1) [16,17,18,19,20,21,22,23,24,25,26,27,28,29,30,31]. This analysis revealed that a significant proportion of these variants (4/12, 33.3%) are located in *SMAD4* intron 2. Among these, the *SMAD4* c.424+5G>A variant, which we molecularly characterized in the present study, was mostly identified in patients with a personal and/or family history of cancer. The other *SMAD4* variants located in intron 2 (i.e., c.425-9A>G, which was identified and molecularly characterized in the present study, c.424+1G>A, and c.425-6A>G) were detected in patients with a family history of JPS. A single *SMAD4* splicing variant (c.667+3G>A) was identified in intron 4. It was found in a patient with breast cancer. Several other *SMAD4* splicing variants (5/12, 41.6%) that occur in intron 8 were identified in patients with a personal and/or family history of JPS. Two of these variants (c.1139G>A and c.1139+1G>A) were previously characterized at the mRNA level and were reported to cause a reading frameshift [16,27]. The remaining *SMAD4* splicing variants (c.1308+1G>A and c.1447+1G>A) identified so far were located in intron 9 and 10, and they were detected in patients with a personal and/or family history of JPS (Figure S2, Table S1).

## 3. Discussion

Genetic variants that disrupt normal patterns of mRNA splicing impair protein synthesis and/or alter protein structure, playing a determining role in disease etiology [32]. It has been reported that a relevant fraction of disease-causative genetic variants (15%–60%) have the potential to alter normal splicing mechanisms [33]. However, the majority of variants involving splice site genomic regions are classified as variants of uncertain significance (VUSs) due to the lack of functional studies on mRNA [34]. As such, investigating the molecular effect of genetic variants that affect the splicing process is crucial to gain insights into disease pathogenesis.

In this study, we expanded the landscape of *SMAD4* splicing variants acting as causative events in JPS etiology and cancer. In particular, we demonstrated that both the c.424+5G>A and the c.425-9A>G *SMAD4* variants, which were identified in two unrelated Italian families, induce aberrant pre-mRNA splicing by creating new cryptic splicing sites and promote the exonization of intronic sequences.

The *SMAD4* gene is located at 18q21.1 and consists of 12 exons and 10 introns, encoding a protein with 552 amino acids and a molecular weight of 60 kDa [35]. The SMAD4 protein contains three domains: the Mad homology 1 (MH1) domain at the N-terminal (aa 14–138), the Mad homology 2 (MH2) domain at the C-terminal (aa 323–552), and the linker region located between the MH1 and MH2 domains (aa 139–322) [35]. SMAD4 has an important role in the TGF-β/SMAD signaling pathway and interacts with other transcription factors to regulate cell proliferation, growth, and differentiation [36].

Loss-of-function somatic alterations of the *SMAD4* gene have been linked to a wide range of tumor types, including pancreatic, colorectal, gastric, breast, and thyroid cancer, and germline genetic alterations, which have been associated with JPS [37,38,39,40,41]. Overall, the majority of *SMAD4* germline variants are frameshift, nonsense, and missense variants, while only a small proportion consists of single or multiexon deletions and splicing variants [11].

The literature review carried out in this study revealed that 12 unique *SMAD4* splicing variants have been reported to date in 24 patients with cancer and/or JPS. Of these variants, only two have been characterized at the mRNA level. In this regard, it is worth noting that the characterization of the molecular effects of *SMAD4* splicing variants may support the clinical diagnosis and management of patients with a personal and/or family history of cancer and/or polyposis.

The *SMAD4* splicing variant c.424+5G>A is located five nucleotides downstream of the splice donor site of intron 2. It was previously described in patients with familial intestinal GC [19], familial pancreatic cancer [21], familial breast cancer [23], and familial colon polyposis/cancer [22]. The patient phenotypes described in these reports do not seem to fulfill the clinical diagnostic criteria of JPS. This variant was also identified in a patient diagnosed with one juvenile polyp and one tubular adenoma of the colon [20]. Due to the lack of sufficient evidence and functional studies investigating its effect at the mRNA level, the clinical assessment of the *SMAD4* c.424+5G>A variant remained inconclusive. Recently, in an effort to identify genomic variants that cause splicing changes using transcriptome sequencing data available in public sequence repositories, Shiraishi et al. considered the *SMAD4* c.424+5G>A variant as a putative pathogenic intron retention associated variant (ppIRAV) [42]. Using in vitro splicing analyses, the authors showed that this variant induces the retention of *SMAD4* intron 2 [42].

In the present study, we identified the *SMAD4* c.424+5G>A variant in a male patient with a personal and family history of CRC. This variant was also detected in his daughter, who reported no clinical manifestations at the time of clinical ascertainment, most likely due to her young age. We experimentally confirmed that the *SMAD4* c.424+5G>A variant promotes the retention of *SMAD4* intron 2 by performing an RT-PCR analysis of patient-derived RNA and an in vitro minigene assay. Moreover, we found that this splicing alteration leads to a reading frameshift and may result in the production of a truncated protein. According to the American College of Medical Genetics and Genomics and Association for Molecular Pathology (ACMG/AMP) guidelines for variant classification, the *SMAD4* c.424+5G>A variant can be classified as pathogenic due to its low frequency in population databases—due to its deleterious effect on the splicing process as predicted by computational tools and its clinical and molecular characterization as performed in the present study—satisfying the following ACMG/AMP pathogenic criteria (PS1, PM2, PM4, PP3, PP4) [43]. Clinically, the phenotype of the patients harboring the *SMAD4* c.424+5G>A variant, as reported in the scientific literature and the present study, does not seem to fulfill the clinical criteria of JPS. A possible explanation as to why this happens may be the lack of an endoscopic evaluation (i.e., small bowel video capsule endoscopy, esophagogastroduodenoscopy) of all the anatomical regions classically involved in JPS. As a result, it might not have been possible to ascertain whether these patients’ colon or stomach tumors originated from juvenile polyps. Anyway, it has recently been reported that not all of the patients carrying a pathogenic variant involving the *SMAD4* or *BMPR1A* genes and submitted to an endoscopic investigation meet the clinical criteria for JPS diagnosis [9]. Current guidelines recommend surveillance programs for patients who fulfill JPS clinical criteria or harbor a germline disease-causative variant in the *SMAD4* or *BMPR1A* genes [44,45]. However, the detection of a VUS in addition to a personal and/or family history of cancer adds to the complexity of clinical surveillance for these patients [46]. Indeed, based on the National Comprehensive Cancer Network guidelines, germline VUSs identified in the *SMAD4* or *BMPR1A* genes in patients without clinical criteria of JPS should not be used to guide medical decision making [45].

The *SMAD4* splicing variant c.425-9A>G is located nine nucleotides upstream of the splice acceptor site of intron 3. To our knowledge, this variant has never been described in the literature before. Based on the ClinVar database, it was previously reported in a patient with a *SMAD4*-related clinical phenotype. In the present study, this variant was identified in a family with clinical manifestations of JPS. In particular, it was detected in two affected brothers, one of which had a pedunculated hamartomatous polyp of the large intestine, while the other developed CRC at 45 years of age. The two brothers inherited the *SMAD4* c.425-9A>G variant from their mother, who also harbored an *APC* pathogenic variant and developed CRC and numerous colon polyps with different histological classifications (hamartomatous, juvenile, and adenomatous). Thus, it can be hypothesized that the clinical phenotype of the affected mother characterized by a colon mixed polyposis was due to the additive effect of the *APC* and *SMAD4* genetic variants, which are associated with the development of adenomatous colon polyps and juvenile colon polyps, respectively. To ascertain the impact of the *SMAD4* c.425-9A>G variant on splicing, total RNA was isolated from the index case, and the *SMAD4* transcript between exon 1 and 4 was amplified by RT-PCR and sequenced. This analysis revealed the retention of eight intronic nucleotides, with a consequent frameshift and the potential production of a truncated protein (p.Asp142Alafs*7). An in vitro minigene assay provided consistent results and confirmed that the *SMAD4* c.425-9A>G variant affects the splicing process. According to ACMG/AMP criteria, our clinical and molecular characterization of this variant provides evidence of pathogenicity, satisfying the following ACMG/AMP pathogenic criteria (PS2, PM2, PM4, PP1, PP3, PP4).

The two *SMAD4* splicing variants identified and characterized in the present study potentially affect the linker domain of the SMAD4 protein. The other two *SMAD4* splicing variants that have been previously characterized at the mRNA level (i.e., c.1308+1G>A and c.1447+1G>A) are located in intron 9 and 10, and they potentially affect the MH2 protein domain [29,31]. Based on previous reports, these are located in the MH2 domain in most patients harboring *SMAD4* pathogenic variants, while they occur in the MH1 and linker domains in a small proportion of patients [9]. However, investigations on genotype–phenotype associations did not identify a clear correlation between genetic variants affecting specific SMAD4 protein domains and clinical heterogeneity in *SMAD4* mutation carriers [11]. In future studies, in-depth molecular characterization of *SMAD4* splicing variants may enable a better understanding of the clinical phenotypes associated with these alterations.

## 4. Materials and Methods

### 4.1. Patient Recruitment

The patients underwent genetic testing after providing informed consent. Molecular testing carried out in this study was based on the routine clinical diagnostic assessment performed at our institute. Written informed consent to perform genetic testing and further studies was obtained from the patients using a form approved by the competent ethics committee, in line with the principles of the Declaration of Helsinki and any other applicable local ethical and legal requirements (protocol code No. 170, date of approval: 31 October 2016).

### 4.2. Genetic Analysis

Genomic DNA was extracted from peripheral blood with the MagCore® Genomic DNA Whole Blood Kit (Amerigo Scientific, New York, NY, USA) according to the manufacturer’s instructions. Then, genomic DNA samples of the index cases of Family 1 and Family 2 were subjected to genetic testing by NGS using a commercial Ion AmpliSeq™ BRCA Reflex—Hereditary Cancer Research Panel (Life Technologies, Carlsbad, CA, USA), which includes 25 hereditary cancer-related genes (*APC*, *ATM*, *BARD1*, *BMPR1A*, *BRIP1*, *CDH1*, *CDK4*, *CDKN2A*, *CHEK2*, *EPCAM*, *MLH1*, *MRE11A*, *MSH2*, *MSH6*, *MUTYH*, *NBN*, *PALB2*, *PMS2*, *PTEN*, *RAD50*, *RAD51C*, *RAD51D*, *SMAD4*, *STK11*, and *TP53*). The analysis was performed as previously described [47]. The clinical classification of the identified genetic variants was performed according to the ACMG/AMP guidelines [43]. The identified genetic variants putatively responsible for the clinical phenotype of the index cases were validated using Sanger sequencing. Segregation analysis was subsequently performed on family members willing to be tested. The genomic region of the *SMAD4* gene was screened for genetic alterations using previously published primer sequences [48]. PCR products were isolated by agarose gel electrophoresis, purified, and sequenced using the BigDye Terminator Cycle sequencing kit (Thermo Fisher, Foster City, CA, USA). All samples were analyzed on a SeqStudio Genetic Analyzer (Thermo Fisher, Foster City, CA, USA). The global population frequency of the identified *SMAD4* variants was retrieved from the 1000 Genome [12], dbSNP [13], gnomAD [49], and NHLBI Exome Sequencing Project [15] databases. Moreover, the HGMD [50], LitVar 2.0 [51], and Clinvar [52] databases were interrogated to assess the pathogenicity of the identified variant.

To evaluate the effect of the *SMAD4* c.424+5G>A and c.425-9A>G variants on RNA splicing, four splice site prediction algorithms integrated into Alamut Visual version 2.15 (Sophia Genetics SAS; Bidart, France) were interrogated simultaneously: Splice Site Finder (SSF), MaxEntScan (MES), Splice Site Prediction by Neural Network (NNS), and Gene Splicer (GS). The default thresholds of each tool were used for the analysis. A variation of more than 10% in at least two algorithms was considered indicative of an effect on the splicing process.

### 4.3. RT-PCR and mRNA Analysis

Total RNA from peripheral blood was extracted with the QIAamp RNA Blood Mini Kit (Qiagen, Hilden, Germany) according to the manufacturer’s instructions. One microgram of RNA was reverse-transcribed to cDNA using the Maxima H Minus First Strand cDNA Synthesis Kit (Life Technologies, Carlsbad, CA, USA). The flanking regions of the *SMAD4* variant sites (NM_005359.6: c.424+5G>A and c.425-9A>G) were amplified using the DreamTaq mastermix (Life Technologies, Carlsbad, CA, USA) and the following primers (10 pmol each): SMAD4_Ex1-2_F AGGCTTCAGGTGGCTGGTC and SMAD4_Ex3-4_R CTTGATGGAGCATTACTCTGCAGT. PCR amplification was carried out at 95 °C for 30 s, followed by 35 cycles at 95 °C for 30 s, 55 °C for 30 s, and 72 °C for 30 s, with a final elongation at 72 °C for 5 min. PCR products were loaded onto 2% agarose gel in 0.5X TBE and visualized using SYBR Safe DNA Gel Stain (Life Technologies, Carlsbad, CA, USA). Sequencing and capillary electrophoresis were performed on a SeqStudio Genetic Analyzer.

### 4.4. Cell Line

The HEK-293 cell line was purchased from ATCC and cultured in DMEM high glucose (HG) without pyruvate (Life Technologies, Carlsbad, CA, USA) and with 10% FBS (Life Technologies, Carlsbad, CA, USA), 1% pyruvate (Life Technologies, Carlsbad, CA, USA), 1% NEAA (Life Technologies, Carlsbad, CA, USA), and 100 U/mL penicillin-streptomycin (Life Technologies, Carlsbad, CA, USA) in a 37 °C and 5% CO2 incubator. The cell line was tested to be mycoplasma-free using the Venor^®^GeM Advance kit (Minerva Biolabs, Berlin, Germany) multiple times throughout this study.

### 4.5. Plasmid Constructs

The pSPL3_SMAD4_Ex2-3_wt and pSPL3_SMAD4_Ex2-3_c.424+5G>A constructs were obtained by amplifying fragments containing *SMAD4* exon 2-3 (NM_005359.6), flanked by upstream (490 nt) and downstream (557 nt) intronic sequences, from the wild-type or mutant allele. Amplification was carried out using the following primers: Cloning_SMAD4_Ex2-3_F_EcoRI CCCATGAATTCGCCCAAGCTGGAGTGCAGT and Cloning_SMAD4__Ex2-3_R_BamHI ACCGATGGATCCAGCTGTGATGGGCATATGCT. The pSPL3_SMAD4_Ex3_wt and pSPL3_SMAD4_Ex3_c.425-9A>G constructs were obtained by amplifying fragments containing *SMAD4* exon 3 (NM_005359.6), flanked by upstream (346 nt) and downstream (327 nt) intronic sequences, from the wild-type or mutant allele. Amplification was carried out using the following primers: Cloning_SMAD4_Ex3_F_EcoRI CCCATGAATTCGGAAGATAGCCCGCGACT and Cloning_SMAD4_Ex3_R_BamHI ACCGATGGATCCTGGCTCTGTGAGATTTTCTTCA. The obtained fragments were cloned into the pSPL3 splicing vector linearized with EcoRI and BamHI. All constructs were confirmed by direct sequencing.

### 4.6. Minigene Assay

HEK-293 cells were transfected for 36 h using Lipofectamine 3000 (Life Technologies, Carlsbad, CA, USA) according to the manufacturer’s instructions. Cells were harvested, and total RNA was extracted with the PureLink™ RNA Mini Kit (Life Technologies, Carlsbad, CA, USA) according to the manufacturer’s instructions and used for RT-PCR to confirm splicing patterns. cDNA was synthesized as described above and used as a template for PCR amplification with the following vector-specific primers: SD6_ FW GTCTGAGTCACCTGGACAACC and SA2_ RV GATCTCAGTGGTATTTGTGAGC. PCR amplification was carried out with Phusion High-Fidelity DNA Polymerase at 98 °C for 30 s, followed by 35 cycles at 98 °C for 10 s, 52 °C for 10 s, and 72 °C for 15 s, with a final elongation at 72 °C for 5 min. PCR products were loaded onto 2% agarose gel in 0.5X TBE and visualized using SYBR Safe DNA Gel Stain. Sequencing and capillary electrophoresis were performed on the SeqStudio Genetic Analyzer.

### 4.7. Literature Review

This literature review was performed using curated databases (HGMD and LitVar 2.0) [50,51] that integrate data from PubMed, PubMed Central Open Access Subset, dbSNP [13], and ClinVar [52], and that enable an accurate search for genetic variants and related human inherited diseases. We searched for any previously identified *SMAD4* splicing variant listed in the ClinVar database. We retrieved all the articles matching the search criteria from the aforementioned databases and collected relevant clinical information (i.e., specific *SMAD4* splicing variant identified; patient genders; age at diagnosis; number of colorectal or gastric polyposis; presence of colorectal, gastric, or other cancer; and family history). Studies including patients without clinical information were excluded.

## 5. Conclusions

In the present work, we provide evidence for the clinical classification of two *SMAD4* splicing variants as pathogenic by using various mRNA analysis approaches. One of these variants (c.424+5G>A) was identified in several patients with a personal and/or family history of cancer. The previous inconclusive classification of this variant may have limited the access to targeted surveillance programs for affected patients while also preventing an in-depth evaluation of the associated phenotypic features. Our study emphasizes that the functional characterization of *SMAD4* splicing variants by means of an RNA analysis is a valuable approach in genetic disease variant interpretation. An accurate classification of *SMAD4* variants based on clinical and molecular evidence is expected to improve the management of patients and their families, leading to more effective screening and/or clinical surveillance strategies. This in turn may help to better decipher the clinical phenotypes associated with these alterations. In conclusion, this study supports the potential of RNA analyses in the clinical classification of *SMAD4* splicing variants in order to ensure tailored genetic counseling, management, and surveillance for patients with a suspected genetic predisposition to GI polyposis and/or cancer.

## Data Availability

The data presented in this study are available in this article.

## Supplementary Materials

The supporting information can be downloaded at: https://www.mdpi.com/article/10.3390/ijms25147939/s1.
