# Supplementary material for: Clinical and Molecular Characterization of SMAD4 Splicing Variants in Patients with Juvenile Polyposis Syndrome
Source: Int J Mol Sci. 2024 Jul 20;25(14):7939. doi: 10.3390/ijms25147939 (PMC11276957; doi:10.3390/ijms25147939)
Supplement: Supplementary file 1 [file ijms-25-07939-s001.zip › Supplementary_Figures_Legend.pdf]

Figure S1: Screenshot from the Alamut software. **(A)** Splicing effect window around *SMAD4* c.424+5G>A splicing variant. The top box represents the *SMAD4* wild-type sequence, while the bottom box represents the *SMAD4* mutated sequence with the c.424+5G>A variant. The dark blue bars represent the predicted splice donor site. The diagram reveals the abolition of the canonical splice donor site at position c.424. All four tools predicted that the identified variant abolishes the canonical splice donor site. **(B)** Splicing effect window around *SMAD4* c.425-9A>G splicing variant. The top box represents the *SMAD4* wild-type sequence, while the bottom box represents the *SMAD4* mutated sequence with the c.425-9A>G variant. The dark blue bars represent the predicted splice acceptor site. The diagram reveals the abolition of the canonical splice donor site at position c.425. All four tools predicted that the identified variant abolishes the canonical splice acceptor site.

Figure S2: Lollipop graph of the *SMAD4* coding sequence showing the distribution of the splicing variants identified to date in patients based on our literature review and on the present study (only patients with clinical information have been included). The clinical manifestations associated with the splicing variants are also reported as colored dots: gastric cancer (green), hamartomatous polyps (light gray), pancreatic ductal adenocarcinoma (orange), breast cancer (pink), Lynch syndrome-associated cancer and/or polyps (light blue), juvenile polyposis syndrome clinical phenotype (black), colorectal cancer (brown) and unknown cancer (white). The scale bar on the left indicates the number of patients carrying each *SMAD4* splicing variant.
