# Supplementary material for: Clinical and Molecular Characterization of SMAD4 Splicing Variants in Patients with Juvenile Polyposis Syndrome
Source: Int J Mol Sci. 2024 Jul 20;25(14):7939. doi: 10.3390/ijms25147939 (PMC11276957; doi:10.3390/ijms25147939)
Supplement: Supplementary file 1 [file ijms-25-07939-s001.zip › Table_S1.pdf]

Table S1: Clinical data and family history of patients with *SMAD4* splicing variants based on our literature review

SMAD4 (NM_005359.6) splicing variant (HGVS)	Chromosome position (GRCh37)	Intron/ exon	Effect on mRNA or protein (HGVS)	Case ID	Gender (age at dx)	No. of colorectal polyps (histology)	CRC (age at dx)	No. of gastric polyps (histology)	GC (age at dx)	Other cancers	Family history (age at dx)	Reference
**c.424+1G>A**	g.48575231G>A	Intron 2		21	F (U)	5 JP<br>(4 HP; 1 AP)					Sister*, JPS<br>Sister*, JPS<br>Maternal aunt*, JPS	[18]
**c.424+5G>A**	g.48575235G>A	Intron 2		P27	M (64)				YES (64)		FDR, GC (66);<br>FDR, HNC (58);<br>SDR, GU (61);<br>SDR, GyC (54);<br>SDR, HepBC (70)	[19]
**c.424+5G>A**	g.48575235G>A	Intron 2			U (38)	2<br>(1 JP; 1 TA)					U	[20]
**c.424+5G>A**	g.48575235G>A	Intron 2			U (U)					PDCA	FDR, PDCA	[21]
**c.424+5G>A**	g.48575235G>A	Intron 2		1092248251	U (U)					LS-C		[22]
**c.424+5G>A**	g.48575235G>A	Intron 2		221	F (<50)					BC	Cancer family history	[23]
**c.424+5G>A**	g.48575235G>A	Intron 2			U (U)							[24]
**c.424+5G>A**	g.48575235G>A	Intron 2	p.Asp142Glyfs*2		M (62)		YES (62)				Brother, CRC<br>Father, CRC<br>Mother, PDCA	Present study
**c.425-9A>G**	g.4857565A>G	Intron 2	p.Asp142Alafs*7		M (35)	3 (HmP, HP)					Brother*, CRC<br>Mother*, CP, CRC	Present study
**c.425-6A>G**	g.48575659A>G	Intron 2	p.I147fsX7	51	F (10)	Numerous		Multiple (AP, HP, JP, PP)			JPS family history	[16]
**c.667+3G>A**	g.48581366G>A	Intron 4			U (U)					BC		[25]
**c.1139G>A**	g.48591976G>A	Exon 8	p.Gly366Alafs*11	44	U (34)	10 (AP)		Hundreds (JP)				[16]
**c.1139+1G>A**	g.48591977G>A	Intron 8		JP102	U (U)							[17]

**c.1139+3A>G**	g.48591979A>G	Intron 8		Case report	F (50)	Multiple (HmP)		Multiple (HmP)	YES		Mother, CRC<br>Maternal uncle, CNSC<br>Maternal cousin, BLCA	[26]
**c.1140-2A>G**	g.48593387A>G	Intron 8	p.Leu381Valfs*12	Case Report	M (13)	Multiple (HmP)					Father, CP, GC<br>Paternal aunt, GC<br>Grandfather, PDCA	[27]
**c.1140-1G>A**	g.48593388G>A	Intron 8		Case report	F (65)			Multiple (HmP, JP)	YES	BC		[28]
**c.1308+1G>A**	g.48593558G>A	Intron 9			U (U)						JPS family history	[29]
**c.1308+1G>A**	g.48593558G>A	Intron 9		12	U (30)	> 15 JP (2 of which AP)						[30]
**c.1447+1G>A**	g.48603147G>A	Intron 10		Case Report	M (28)	Multiple (HmP)	YES (28)	Multiple (HP)			Father, 7 HP, 8 AP<br>Daughter, CP (JP)<br>Son, JP colon	[31]

HGVS, Human Genome Variation Society; dx, diagnosis; F, female; M, male; CRC, colorectal cancer; GC, gastric cancer; AP, adenomatous polyp; CP, colon polyp; JP, juvenile polyp; HP, hyperplastic polyp; HmP, hamartomatous polyp; PP, inflammatory pseudopolyp; TA, tubular adenoma; JPS, juvenile polyposis syndrome; FDR, first-degree relative; SDR, second-degree relative; BC, breast cancer; BLCA, bladder cancer; CNSC, central nervous system cancer; HepBC, hepatobiliary cancer; HNC, head and neck cancer; GyC, gynecological cancer; GU, gastric ulcer; LS-C, Lynch syndrome associated cancer; PDCA, pancreatic ductal adenocarcinoma; U, unknown (no information available); *, individuals carrying the *SMAD4* splicing variant.;
