# Supplementary figures and images for: Clinical and Molecular Characterization of SMAD4 Splicing Variants in Patients with Juvenile Polyposis Syndrome
Source: Int J Mol Sci. 2024 Jul 20;25(14):7939. doi: 10.3390/ijms25147939 (PMC11276957; doi:10.3390/ijms25147939)

A

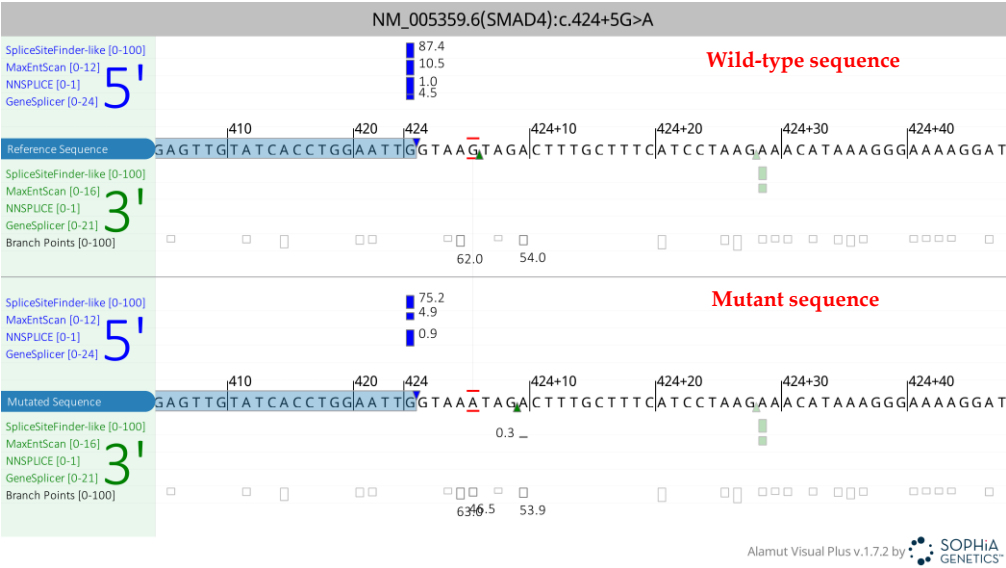

|               | SSF<br>[0-100] | MES<br>[0-12] | NNS<br>[0-1] | GS<br>[0-24] |
|---------------|----------------|---------------|--------------|--------------|
| Threshold     | ≥ 70           | ≥ 0           | ≥ 0.4        | ≥ 0          |
| Exon 2- c.424 | 87.4 → 75.2    | 10.5 → 4.9    | 1 → 0.9      | 4.5 → -      |

B

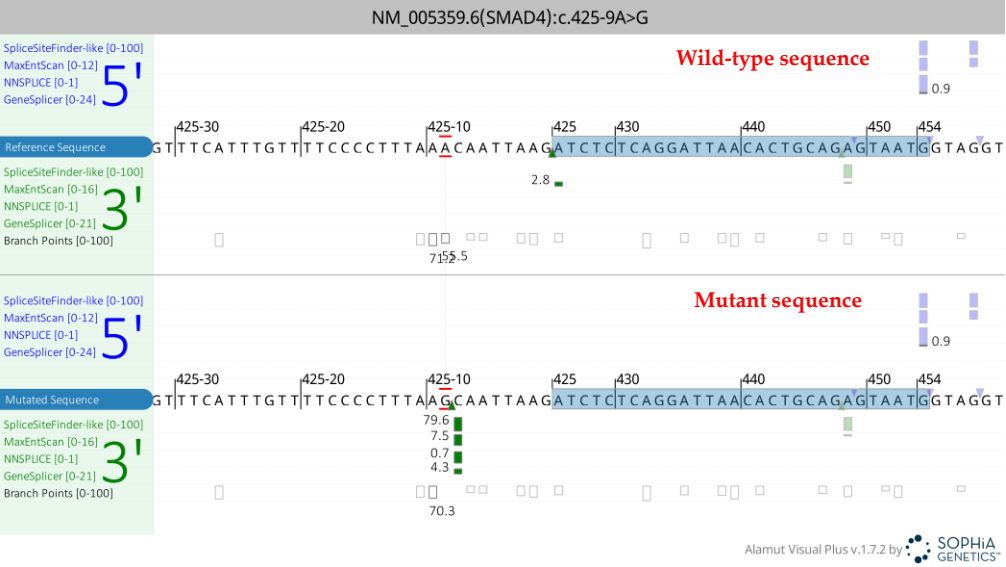

|                 | SSF<br>[0-100] | MES<br>[0-12] | NNS<br>[0-1] | GS<br>[0-24] |
|-----------------|----------------|---------------|--------------|--------------|
| Threshold       | ≥ 70           | ≥ 0           | ≥ 0.4        | ≥ 0          |
| Intron 2- c.425 | - → 79.6       | 2.8 → 7.5     | - → 0.7      | - → 4.3      |

Supplement: Supplementary file 1 [file ijms-25-07939-s001.zip › Supplementary_Figure_1.pdf]

Figure S2

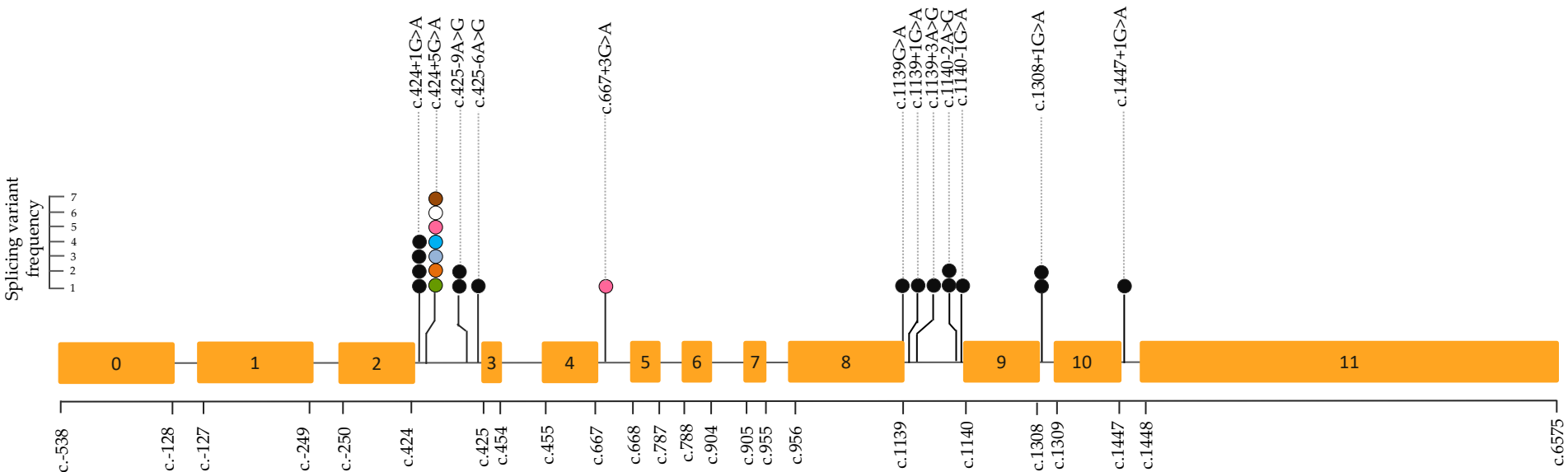

SMAD4 (NM\_005359)

Supplement: Supplementary file 1 [file ijms-25-07939-s001.zip › Supplementary_Figure_2.pdf]
